# Age Differences in the Consumption and Avoidance of COVID-19 Information

**DOI:** 10.1093/geroni/igaa057.3418

**Published:** 2020-12-16

**Authors:** Stephanie Deng, Julia Nolte, Corinna Loeckenhoff

**Affiliations:** Cornell University, Ithaca, New York, United States

## Abstract

Staying informed about COVID-19 is crucial to maintaining public health. Although older adults are at increased risk of complications, recent data (Global Web Index, 2020) suggest that they are less likely to seek out information about COVID-19. This is consistent with prior evidence indicating that information seeking is negatively associated with age (Mata & Nunes, 2010). However, it remains unclear whether older adults merely fail to seek information or intentionally avoid information. In response, we examined whether age is associated with general information seeking and deliberate information avoidance in the wake of the COVID-19 pandemic. Based on previous work indicating age-related shifts in motivational priorities (Carstensen, 2006) we also examined whether avoidance motives differ by age. In a pre-registered online study, an adult lifespan sample (N=500, Mage=49.90, 51% female, 67% non-Hispanic White) completed self-report measures of media consumption, information avoidance, and avoidance motives with respect to the COVID-19 pandemic. In addition, we measured behavioral information avoidance by allowing participants to opt out of receiving valid COVID-19 information. As predicted, age was associated with decreased media consumption (p<.001) and higher information avoidance about COVID-19 on the behavioral measure (p<.01). Self-reported information avoidance, in contrast, was highest among younger adults (p<.05). Further, with the exception of concerns about trustworthiness, older adults were less likely than younger adults to endorse various information avoidance motives (ps<.05). Thus, although information seeking is lower and behavioral information avoidance about COVID-19 is higher in later life, this cannot be traced to explicit intentions or select motives.

